# A metaheuristic automated framework for quality improvement of CT imagery

**DOI:** 10.1038/s41598-026-54389-0

**Published:** 2026-05-24

**Authors:** S. Ramasamy, Justin Joseph, V. R. Simi

**Affiliations:** Manipal Institute of Technology Bengaluru, Manipal Academy of Higher Education, Manipal, 576104 India

**Keywords:** Computed tomography, Contrast-limited adaptive histogram equalization, Perception-based image quality evaluator, Whale optimizer, Computational biology and bioinformatics, Engineering, Mathematics and computing

## Abstract

Contrast-limited adaptive histogram equalization (CLAHE) is a technique often used for enhancing the quality of computed tomography (CT) images. The quality of enhanced CT slices produced by the CLAHE depends on the selection of the clip-limit (CL). The CLAHE driven with inadequate value of CL selected via trial and error may amplify noise, mask subtle structures, and hinder the overall perceptual quality of the processed CT slices. As a solution to this problem, we introduce a metaheuristic framework to facilitate automated CL selection in CLAHE, especially for CT contrast enhancement. The framework uses the whale algorithm as optimizer and a perception-based image quality evaluator (PIQE) as fitness. On 315 CT slices, the WOA-PIQE-CLAHE framework produced outputs that have lower PIQE (29.8063 ± 1.1433) and higher contrast (71.5385 ± 1.0408) compared to the low-contrast slices (PIQE = 32.0184 ± 0.9894 & Contrast = 69.3130 ± 1.0465). Increase in contrast and decrease in PIQE manifest that the framework improves tissue contrast without hindering the perceptual quality of the CT slices.

**Supplementary Information:**

The online version contains supplementary material available at 10.1038/s41598-026-54389-0.

## Introduction

### Background

**Fig. 1:**
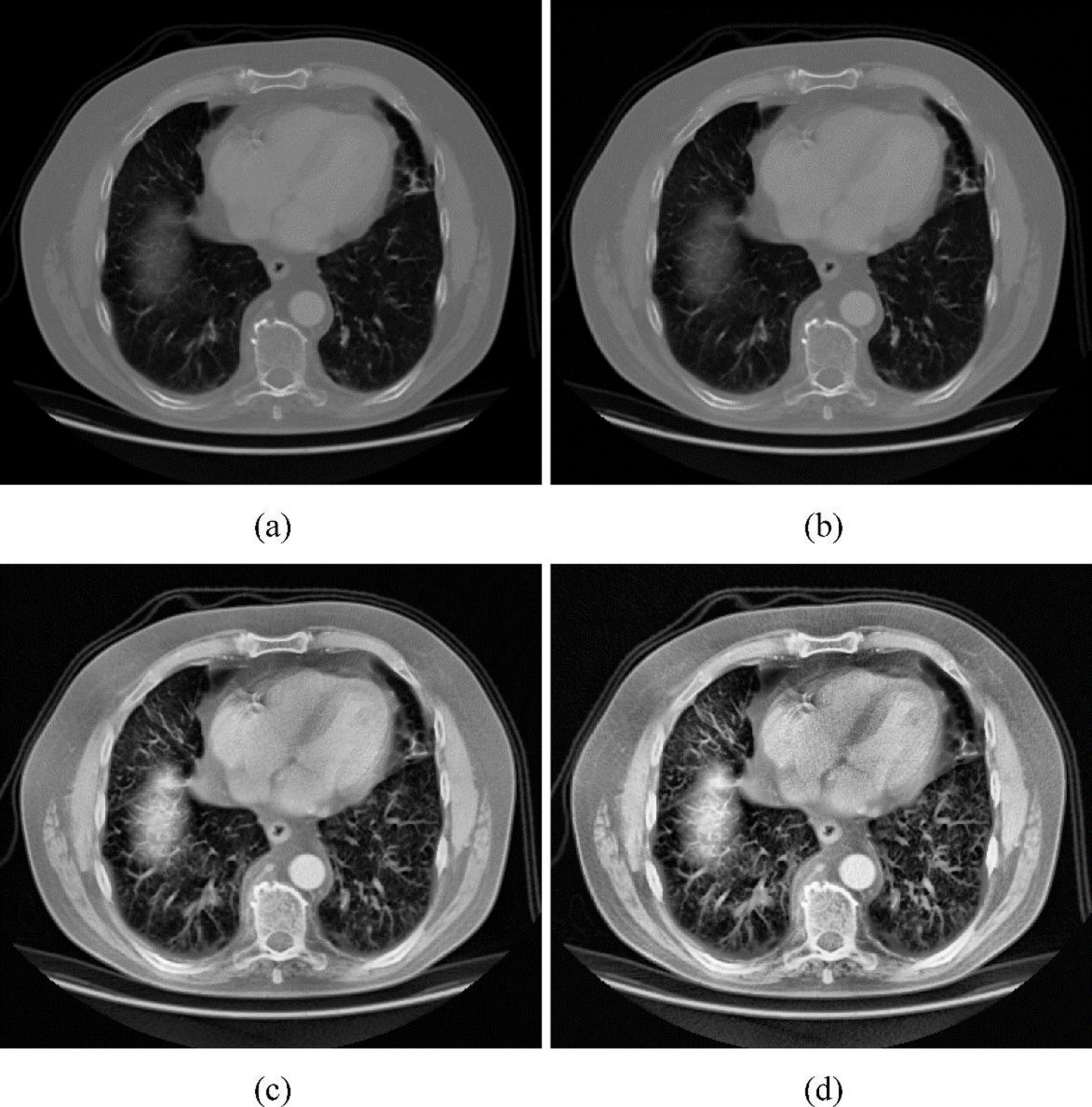
Impact of the selection of CL on the quality of an enhanced CT slice produced by the CLAHE (**a**) Low-contrast CT slice (**b**) CLAHE output (O/P) at CL = 0.0001 (**c**) CLAHE O/P at CL = 0.01 (**d**) CLAHE O/P at CL = 0.02.

Contrast-limited adaptive histogram equalization (CLAHE) is widely used for improving the quality of computed tomography (CT) images^1–5^. Quality of enhanced CT slices produced by the CLAHE depends on the value of clip-limit (CL). The CL is a user-defined parameter that needs to be tuned manually. Impact of the selection of CL on the quality of an enhanced CT slice produced by the CLAHE is illustrated in Fig. 1. Inadequate values of CL selected via trial and error hinders the perceptual quality of processed CT slices. A low CL results in absolutely no or minimal enhancement, as seen in Fig. 1b. A high CL amplifies noise and obscures subtle textural information as seen in Fig. 1c,d. Hence, algorithms that facilitate automated selection of image-adaptive CL are required.

### Related methods

Two strategies are generally adopted in the literature to automate the selection of the parameters of image processing algorithms. The first strategy is to deploy pre-trained deep learning image-to-scalar regression models (DLISRM) that can predict the optimum parameters of the image processing algorithms from the images themselves. The EfficientNet DLISRM developed by Venugopal et al.^6^ to predict the optimum parameter setting of a modified sigmoid transform used for enhancing the quality of dermatological macro-images is an example of the first strategy. The DLISRM needs a large set of images and corresponding optimum parameters of the image processing algorithms for training them.

The second strategy uses nature-inspired metaheuristic algorithms and appropriate fitness functions to automate the selection of the parameters of image processing algorithms. Generally, image quality assessment metrics (IQAMs) or similar measures are used as fitness functions in such metaheuristic frameworks. For example, Tamilarasan and Rajamani^7^ adopted snake optimizer and fuzzy entropy fitness for optimizing the multilevel thresholds for segmenting natural-scene images. Luo et al.^9^ used an improved version of the Hunger games search (HGS) optimizer and one of the IQAMs, namely the structural similarity index metric (SSIM) or normalized mutual information (NMI), to find the optimal translation, scaling, and shearing parameters to improve the performance of multimodality medical image co-registration. The approaches mentioned above^7,9^ have used IQAMs as fitness functions. Unlike this, there are approaches in which the performance indices of machine learning models that do the final clinical decision-making are used as fitness functions. For example, Guneş and Kalkan^8^ employed the genetic algorithm (GA) to optimize the relative weights of the colour channels used in a colour-to-grayscale conversion model. The accuracy of image categorization exhibited by one of the classifiers, namely support vector classifier (SVC), logarithmic linear classifier (LOGLC), and quadratic discriminant classifier (QDC), was used as the fitness. Such fitness functions and optimization frameworks just give a common optimum parameter value for all images in the dataset, rather than individualized optimum values per image.

In addition to the parameter optimization of image segmentation, registration, and decolourization algorithms discussed above, metaheuristic algorithms are used in the literature for parameter optimization of image enhancement algorithms also. For example, Almero et al.^10^ used the GA and entropy fitness to optimize the selection of parameters of the dark channel prior dehazing algorithm on underwater images. Bi et al.^11^ also used the GA for optimizing the underwater image dehazing. The accuracy of object detection was used as fitness. Devi et al.^12^ used the RIME optimization algorithm (ROA) and the gradient singular value decomposition (GSVD) IQAM as fitness for selecting the gamma correction parameters employed for devignetting wireless capsule endoscopy (WCE). Elewi et al.^13^ used the grey wolf optimizer and contrast enhancement-based contrast-changed image quality (CEIQ) IQAM as fitness to optimize the incomplete beta function (IBF) parameters for natural-scene image enhancement. Manongga et al.^14^ used the patch-based contrast quality index (PCQI) as fitness to identify the optimum weight of the linear combination that combines low-contrast natural-scene images and the respective histogram equalization (HE) outputs (O/Ps). In spite of the automated dehazing, devignetting, IBF, etc. discussed above, metaheuristic methods for optimizing the CLAHE parameters are very rare in the literature. Quintana et al.^15^ recently used a multi-objective cuckoo search (MOCS) metaheuristic optimization algorithm (MOA) for selecting CL parameter of the CLAHE. The contrast and standard deviation (std) of noise were used as fitness functions. A CLAHE O/P with high contrast and low noise does not guarantee preservation of information content and natural appearance.

### Motivation and objectives

Most of the metaheuristic algorithms in the literature are mainly used in the image processing domain for parameter optimization of image segmentation, registration, decolourization, dehazing, and devignetting algorithms. Even though certain other applications for automating the IBF-based image enhancement do exist, a framework devoted to automating the selection of CL in the CLAHE deployed for the CT enhancement task is not available. Noise std and contrast used as finesses in the multi-objective frameworks^15^ for automating the CL selection do not comprehensively cover all aspects of perceptual quality. It is difficult to have a single optimal solution in the case of multi-objective optimization, especially when features like high contrast and low noise are conflicting with each other. Improving the contrast will amplify noise as well. Multi-objective optimization gives only a set of Pareto optimal solutions. Increased complexity of multi-objective optimization is another concern. To address these issues, we introduce a metaheuristic framework for automated selection of CL for contrast enhancement applications in CT. The framework uses the whale algorithm as optimizer and a perception-based image quality evaluator (PIQE) as fitness.

## Methodology

### CLAHE algorithm

The CLAHE parses the input (I/P) CT slice into distinct tiles with no overlap and processes them individually^16^. Thus, a CT slice with $\:R\times\:C$ dimension is parsed into discrete tiles, each having $\:\left(R/{S}_{R}\right)\times\:\:\left(C/{S}_{C}\right)$ pixels in it. Typical choice of the scaling factors is $\:{S}_{R}={S}_{C}=8$^16^. The most influential parameter supplied to the CLAHE is a normalized CL $\:\alpha\:$. The CLAHE algorithm computes a final clipping threshold (FCT) $\:\tau\:$ internally from the normalized CL $\:\alpha\:$, and the total number of intensity bins (TNIB) $\:{N}_{I}$ used for computing the histogram $\:h\left(n\right),n=\mathrm{0,1},2,\dots\:\dots\:,{N}_{I}$, and the number of pixels in an individual tile $\:RC/{S}_{R}{S}_{C}$. The FCT thus computed is^16^,

1 $$\tau = \left\lceil {RC/S_{R} S_{C} N_{I} } \right\rceil + O\left( {\alpha \left( {\left( {RC/S_{R} S_{C} } \right) - \left\lceil {RC/S_{R} S_{C} N_{I} } \right\rceil } \right)} \right)$$

In (1), $\left\lceil \cdot \right\rceil$ is a ceiling operator and $\:O\left(.\right)$ indicates numerical round-off. The term $\left\lceil RC/{S}_{R}{S}_{C}{N}_{I} \right\rceil$ in (1) is the amplitude of a uniform histogram. The default selection of TNIB in (1) is $\:{N}_{I}=256$. Following the computation of the FCT, the histogram $\:h\left(n\right),n=\mathrm{0,1},2,\dots\:\dots\:,{N}_{I}$ of the pixel values in the tile are computed. Total number of pixels that are overflowing FCT, $\:{N}_{O}(n=0)$, average number of overflowing pixels $\:{\mu\:}_{O}$, and the upper limit for histogram amplitude $\:{h}_{u}$ are then computed from the histogram using (2)^16^.

2 $$N_{O} (n = 0) = \sum\limits_{{n = 0}}^{{N_{I} }} {\max \left\{ {\left( {h\left( n \right) - \tau } \right),0} \right\}} ,\mu _{O} = \left\lfloor {T\left( {n = 0} \right)/N_{I} } \right\rfloor ,\:and\ h_{u} = \:\tau \: - \:\mu \:_{O}$$

In (2), $\left\lfloor \cdot \right\rfloor$ is a floor operator. Making use of the average overflow $\:{\mu\:}_{O}$ and upper histogram amplitude limit $\:{h}_{u}$, the content of each histogram bin $\:h\left(n\right),n=\mathrm{0,1},2,\dots\:\dots\:,{N}_{I}$ is then modified based on the conditions in expression (4)^16^.

3 $$\:h\left(n\right)=\:\left\{\begin{array}{cc}\tau\:&\:if\ h\left(n\right)>\:{h}_{u}\:\\\:h\left(n\right)+{\mu\:}_{O}&\:Otherwise\end{array}\right.$$

The total overflow $\:T\left(n\right)$ is updated by incorporating the changes that happened on the histogram during the modification process in (3), as (Karel, 1994),

4 $$\:{N}_{O}\left(n\right)=\:\left\{\begin{array}{cc}{N}_{O}\left(n-1\right)&\:if\  h\left(n\right)>\tau\:\:\\\:{N}_{O}\left(n-1\right)-\:\left(\tau\:-h\left(n\right)\right)&\:if\ {h}_{u}<h\left(n\right)\le\:\tau\:\\\:{N}_{O}\left(n-1\right)-{h}_{u}&\:Otherwise\end{array}\right.$$

At the end of the update, $\:{N}_{O}(n={N}_{I})$ reflects the final value of the remaining number of pixels overflowing the FCT. Residue pixels $\:T\left({n=N}_{I}\right)$ are then recursively put back into the histogram bins $\:h\left(n\right),n=\mathrm{0,1},2,\dots\:\dots\:,{N}_{I}$ at a rate of one pixel. Major refilling of the histogram bins happens during the modification step described in (3). Only the remaining residue is emptied during the recursive allocation. Enhanced intensity $\:E\left(n\right)$ corresponding to an arbitrary intensity *\:n* in the tile is computed using the formulation in (5) that involves the desired upper intensity bound $\:{I}_{u}$, lower intensity bound $\:{I}_{l}$, TNIB $\:{N}_{I}$, and cumulative histogram $\:H\left(n\right)$ (Karel, 1994).

5 $$\:E\left(n\right)=min\left\{\left({I}_{l}+\:\left(H\left(n\right)\left(\left({I}_{u}-{I}_{l}\right)/{N}_{I}\right)\right)\right),{I}_{u}\right\}$$

After applying the enhancement procedure to individual tiles, the resultant enhanced tiles are stitched together. Pixel values at tile intersection points are recomputed from the neighbouring pixels using bilinear interpolation to avoid blockiness artefacts caused by the tile-wise processing involved in the CLAHE.

### Whale optimization algorithm

We have used the WOA^17^ to automate the selection of the CL in the CLAHE. The formulation of the WO is based on the social behaviour of humpback whales, especially the bubble-net hunting^17^. In the initial phase (*\:t=0*) of the WO, an array *\:W* of *\:N* random whale positions $\:{w}_{n},\:n=\mathrm{1,2},3,\dots\:.\:\dots\:.,N,\:{w}_{n}\in\:W$ are generated within the permitted range $\:\left[{w}_{l}\:{w}_{u}\right]$ of the parameter to be optimized. In the first phase, values in the array greater than $\:{w}_{u}$ are downscaled to $\:{w}_{u}$ and values less than $\:{w}_{l}$ are up-scaled to $\:{w}_{l}$. In the third phase, the fitness function values $\:{\varphi\:}_{n},\:n=\mathrm{1,2},3,\dots\:\dots\:,N$ are computed for each whale position $\:{w}_{n}$ one by one and compared with the lowest fitness value seen so far $\:{\varphi\:}_{min}$ (score of the leader whale). In the fourth phase, the leader whale position $\:\omega\:$ is replaced by $\:{w}_{n}$ and the score of the leader whale $\:{\varphi\:}_{min}$ is replaced by $\:{\varphi\:}_{n}$ if $\:{\varphi\:}_{n}<{\varphi\:}_{min}$. In the fifth phase, whale positions are updated according to (6). The updated whale position $\:{w}_{n}\left(t\right)$ at $\:{t}^{th}$ iteration is^17^,

6 $$\:{w}_{n}\left(t\right)=\:\left\{\begin{array}{cc}{w}_{k\:}\left(t-1\right)-T\left(\left|2{r}_{2}{w}_{k}\left(t-1\right)-{w}_{n}\left(t-1\right)\right|\right)&\:if\ {r}_{4}<0.5\ \&\:\left|T\right|\:\ge\:1\\\:\omega\:-T\left(\left|2{r}_{2}\omega\:-{w}_{n}\left(t-1\right)\right|\right)&\:if\ {r}_{4}<0.5\ \&\:\left|T\right|\:<1\\\:\left(\left|\omega\:-{w}_{n}\left(t-1\right)\right|\right){e}^{Ucos\left(2\pi\:U\right)}+\:\omega\:&\:if\ {r}_{4}\ge\:0.5\end{array}\right.$$

given $k = \left\lfloor {N\{ r\} \_\{ 5\} + 1} \right\rfloor$. $\:{w}_{k}\left(t-1\right)$ indicates a random whale position fetched from the array at $\:\left(t-1\right)$ iteration. In (6), the terms *\:T* and *\:U* are^17^,

7 $$\:T=\:\left(2{r}_{1}-1\right)\left(2-\frac{2t}{{t}_{max}}\right)\:and\ U=\:{r}_{3}\left(-2-\frac{t}{{t}_{max}}\right)+1$$

**Fig. 2:**
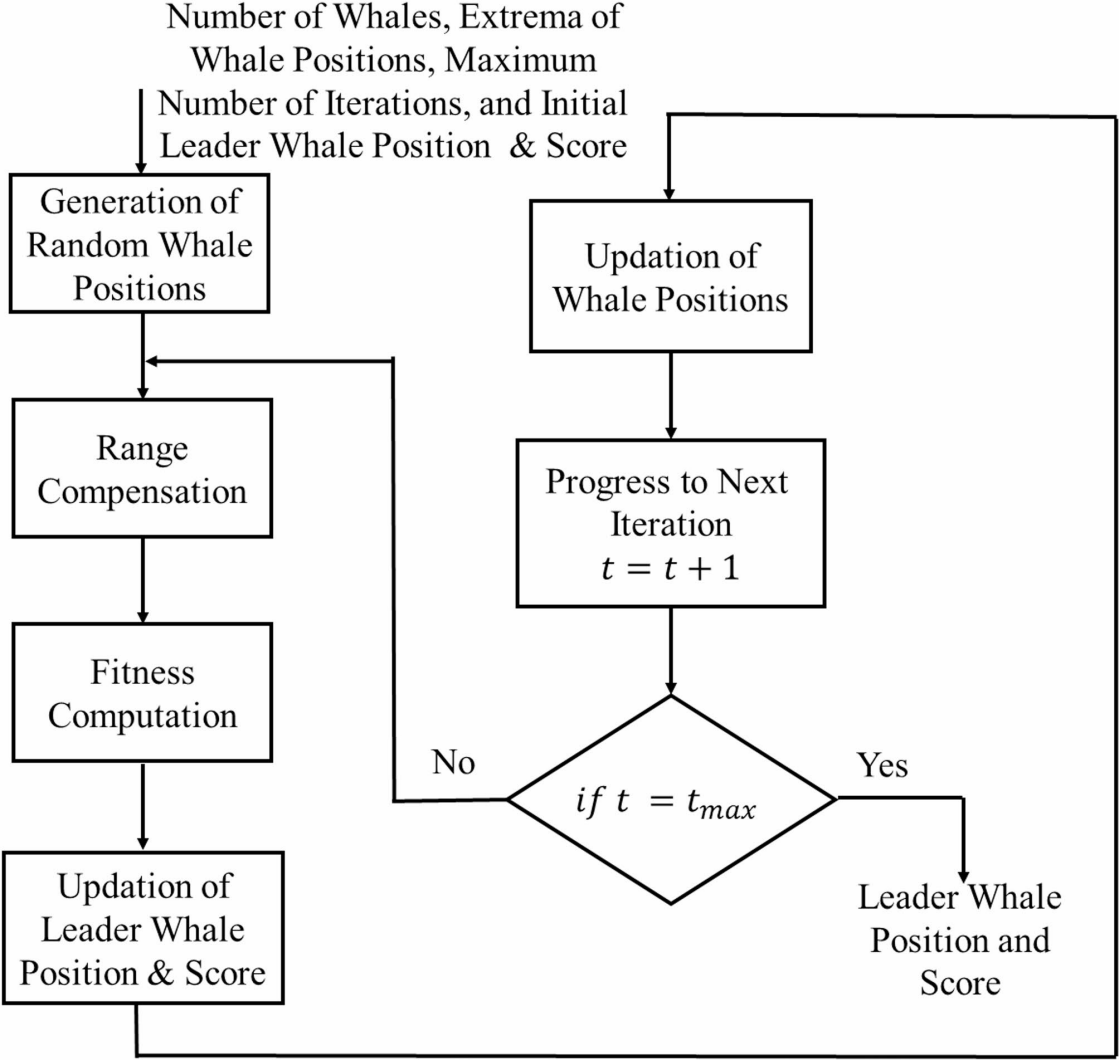
A schematic demonstrating key computing phases of the whale optimizer.

The index of the current iteration *\:t* is incremented by one, and the second to fifth phases are repeated while *\:t* is greater than the user-defined value of the maximum number of iterations $\:{t}_{max}$. $\:{r}_{m},\:m=\left\{\mathrm{1,2},\mathrm{3,4},5\right\}$ are random values within [0 1]. The same procedure is adopted across different dimensions when the number of parameters to be optimized is more than 1. The PIQE score of the enhanced CT slice obtained from the CLAHE is the fitness in our context. We have set *\:N=30* and $\:{t}_{max}=30$. The limit of the CL is set as [0.00001 0.02]. A schematic demonstrating key computing phases of the whale optimizer is shown in Fig. 2.

### Perception-based image quality evaluator

We use a perception-based image quality evaluator (PIQE)^18^ as fitness. For estimating the PIQE, a matrix of mean-subtracted contrast-normalised coefficients (MSCNCs) is computed from the CLAHE O/P. Statistics of MSCNCs reflect the impacts of noise, blockiness, and blur distortions more meaningfully than that of the pixel values^18^. The MSCNC matrix is then divided into blocks, each comprising 16 × 16 pixels, without overlap. Only *\:K* spatially active blocks (SABs) $\:{B}_{k},\:k=\mathrm{1,2},3,\dots\:,K$ with MSCNC variance greater than 0.1 are further considered for computing the PIQE. Thus, an arbitrary MSCNC in a SAB $\:{B}_{k}$ is $\:{b}_{k}\left(r,c\right),\:r=\mathrm{1,2},3,\dots\:\dots\:,16\ and\  c=\mathrm{1,2},3,\dots\:\dots\:,16$. Boundary rows and columns of each SAB are divided into 11 segments, each carrying 6 consecutive MSCNCs. A total of 44 segments is available in each SAB. For example, the set of the MSCNCs that constitute $\:{l}^{th}$ segment of the first boundary row of $\:{B}_{k}$ is $\:\left\{{b}_{k}\left(1,l\right),{b}_{k}\left(1,l+1\right),{b}_{k}\left(1,l+2\right),\:{b}_{k}\left(1,l+3\right),{b}_{k}\left(1,l+4\right),{b}_{k}\left(1,l+5\right)\right\},\:\:l=\mathrm{1,2},3,\dots\:\dots\:,11$. Then the std of the MSCNCs in each segment is computed. Following this, a decisive parameter value $\:\vartheta\:$ is computed from the stds of the MSCNCs in the two middle columns of the SAB (8th and 9th ) $\:{\sigma\:}_{1}$, the rest of the columns $\:{\sigma\:}_{2}$, and the whole SAB $\:\sqrt{{v}_{k}}$ as^18^,

8 $$\:\vartheta\:=\:\frac{\left({\sigma\:}_{1}/{\sigma\:}_{2}\right)-\:\sqrt{{v}_{k}}}{max\left\{\left({\sigma\:}_{1}/{\sigma\:}_{2}\right),\sqrt{{v}_{k}}\right\}}$$

$\:{v}_{k}$ is the variance of the MSCNCs in $\:{B}_{k}$. Final PIQE is computed from the distortion scores $\:{Z}_{k},\:k=\mathrm{1,2},3,\dots\:,K$ of individual SABs. Distortion score $\:{Z}_{k}$ of the SAB $\:{B}_{k}$ is set to $\:{1-v}_{k}$ if std of MSCNCs in any of the boundary segments in $\:{B}_{k}$ is less than 0.1 and $\:\sqrt{{v}_{k}}\:\le\:2\vartheta\:$. The score $\:{Z}_{k}$ is set to $\:{1-v}_{k}$ if stds of MSCNCs in all boundary segments in $\:{B}_{k}$ are greater than or equal to 0.1 and $\:\sqrt{{v}_{k}}>2\vartheta\:$. The score is set to 1 if the std of MSCNCs in any of the boundary segments in $\:{B}_{k}$ is less than 0.1 and $\:\sqrt{{v}_{k}}>2\vartheta\:$. The distortion score is set to zero if both conditions are not met. Final PIQE score is^18^,

9 $$\:PIQE=\frac{1}{K+1}\left(\left(\sum\:_{k=1}^{K}\:{Z}_{k}\right)+1\right)$$

**Fig. 3:**
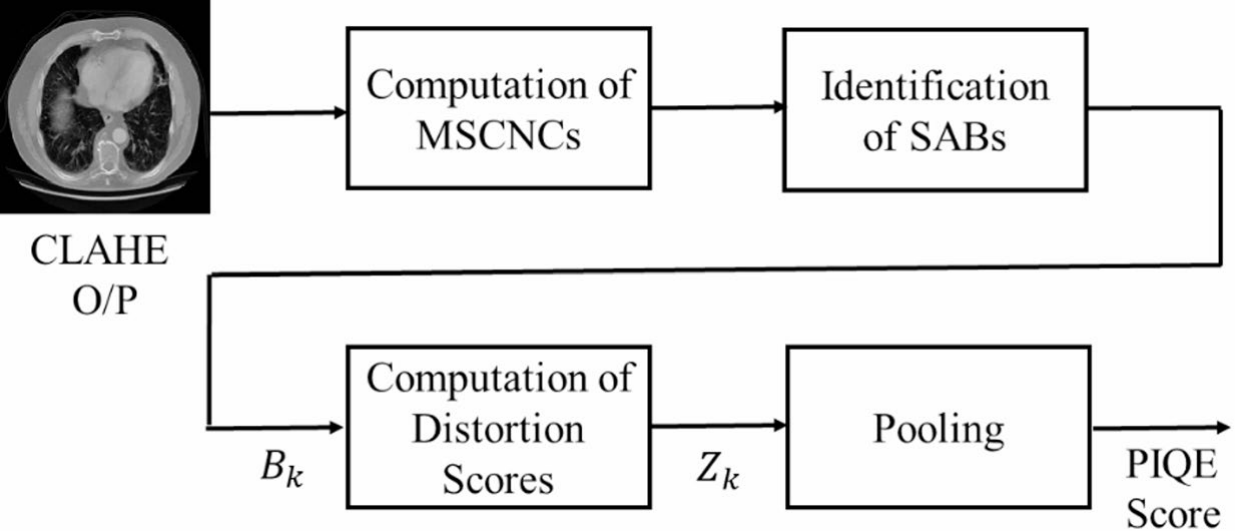
A schematic demonstrating key computing phases of the PIQE.

A schematic demonstrating key computing phases of the PIQE is shown in Fig. 3.

## Results and discussion

**Fig. 4:**
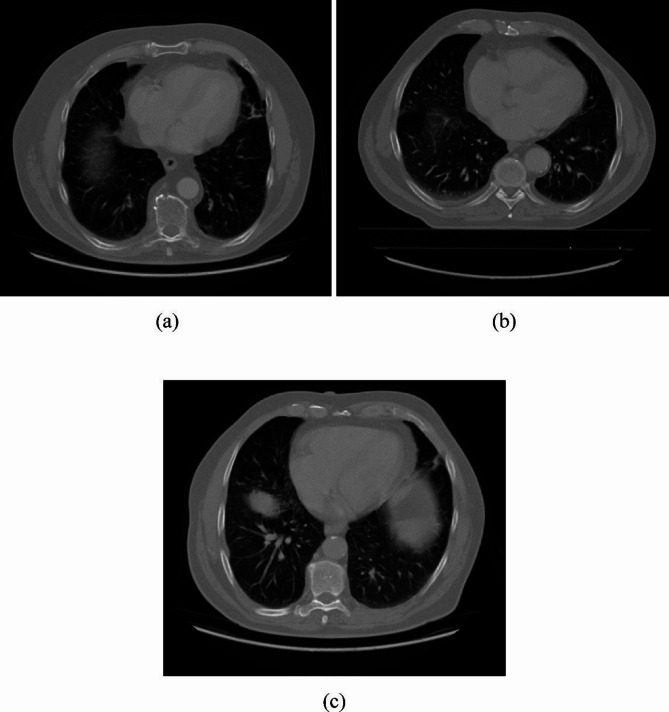
Representative CT slices (**a**) CT slice 1 (**b**) CT slice 2 (**c**) CT slice 3.

We have used 315 CT slices collected from an open-access dataset for validating the WOA-PIQE-CLAHE framework for automating the selection of CL. The dataset is available for free download at https://www.kaggle.com/datasets/borhanitrash/lung-cancer-ct-scan-dataset. It contains 315 CT slices. The slices belong to four different classes. The classes are 120 slices of adenocarcinoma patients, 51 slices of large cell carcinoma patients, 90 slices of squamous cell carcinoma and 54 slices of healthy controls. The dataset is often used for training the deep learning models used for characterizing the type of lung carcinoma from CT images^19^. Three representative CT slices that we have used to demonstrate the performance of the WOA-PIQE-CLAHE framework are shown in Fig. 4.

### Validation of fitness

We compare the suitability of PIQE as a fitness for facilitating the selection of the CL, with visual information fidelity (VIF), GSVD, CEIQ, and PCQI. These IQAMs are chosen for comparison, considering their prior applications as fitness functions to optimize the parameters of image enhancement algorithms. For example, VIF is an IQA recently used by Rashmi & Periyasamy^20^ as the fitness to automate the parameter selection of CLAHE on radiographs. The GSVD metric is a fitness used by Devi et al.^12^ for optimizing the gamma correction of WCE. The CEIQ measure^13^ has already been used as a fitness to optimize the parameters of IBF parameters. Similarly, the PCQI is already used by Manongga et al.^14^ to identify the optimum weight of the linear combination that combines low-contrast natural-scene images and the respective HE O/Ps.

**Fig. 5:**
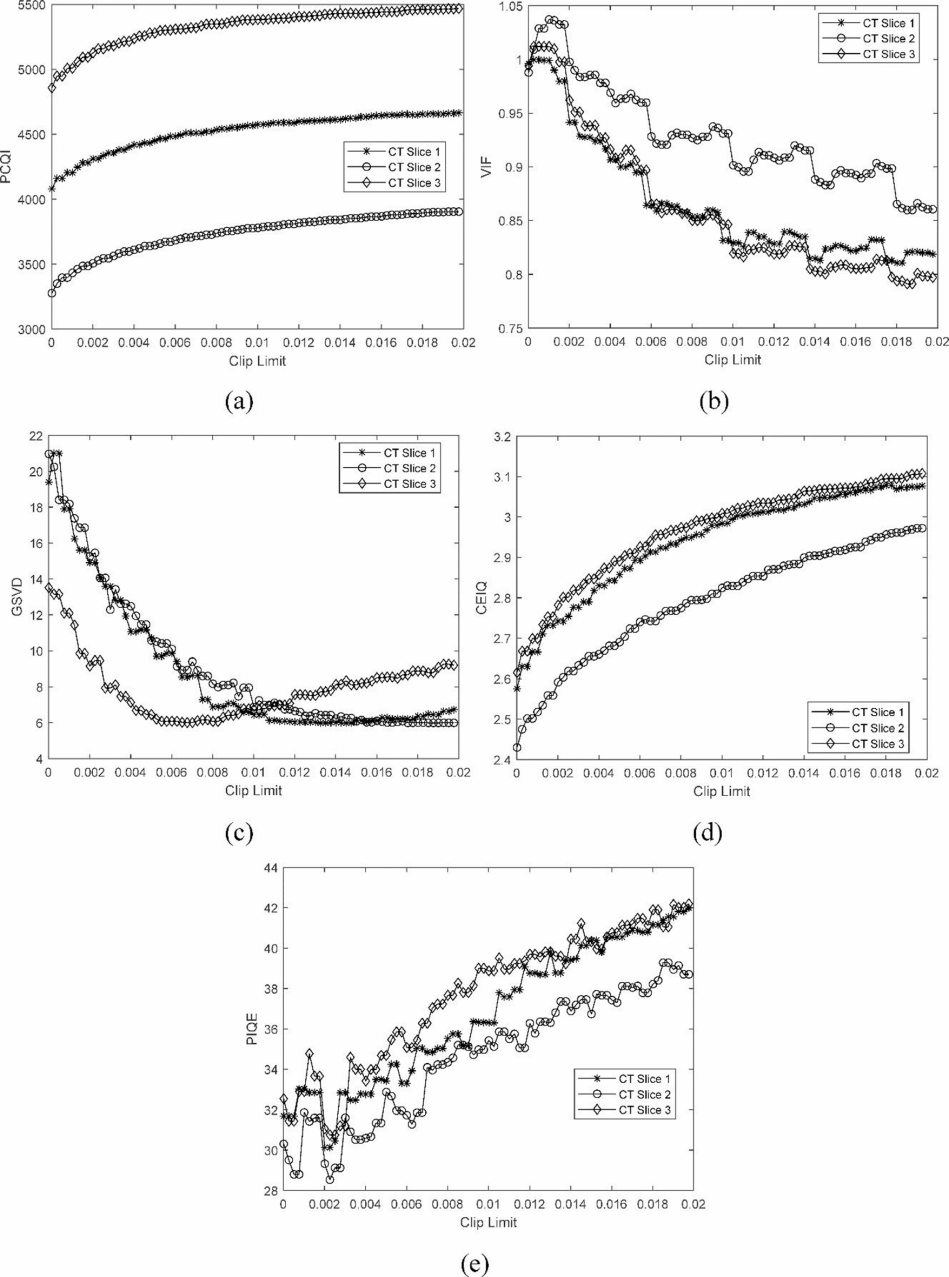
Plots of fitness functions versus CL (**a**) PCQI (**b**) VIF (**c**) GSVD (**d**) CEIQ (**e**) PIQE.

Plots of IQAMs used as fitness functions versus CL on three representative CT slices depicted in Fig. 4 are shown in Fig. 5. Both PCQI (Fig. 5a) and CEIQ (Fig. 5d) are continuously increasing on all three CT slices. It is already shown in Fig. 1d that, for CL values equal to or above 0.02, the CLAHE amplifies noise and obscures subtle textural information on the enhanced CT slices. Hence, PCQI and CEIQ are not good choices as fitness functions. In Fig. 5c, the GSVD has a unique global minimum point on CT slice 1. This is true on CT slice 3 also. However, on the CT slice, the GSVD is continuously falling without leaving any global minimum point. In addition, as a fitness, the GSVD does not have a common pattern among different CT slices. These two aspects preclude the suitability of the GSVD as a fitness function in the context of CLAHE-based CT enhancement. In Fig. 5b, on CT slice 2 and CT slice 3, the VIF remains constant for a narrow range of CL values. The best value of CL at which the PIQE is minimum in Fig. 5e is within the narrow range of the CL where the VIF is maximum in Fig. 5b on each CT slice. However, the VIF as a fitness is not very sensitive to the variations in the CL, as is the PIQE. PIQE is responsive enough even to fine variations of the CL. PIQE has a unique global minimum for each CT slice. These two attributes of the PIQE manifest its suitability as a fitness function in the context of CLAHE-based CT enhancement.

### Validation of optimization algorithm

**Fig. 6:**
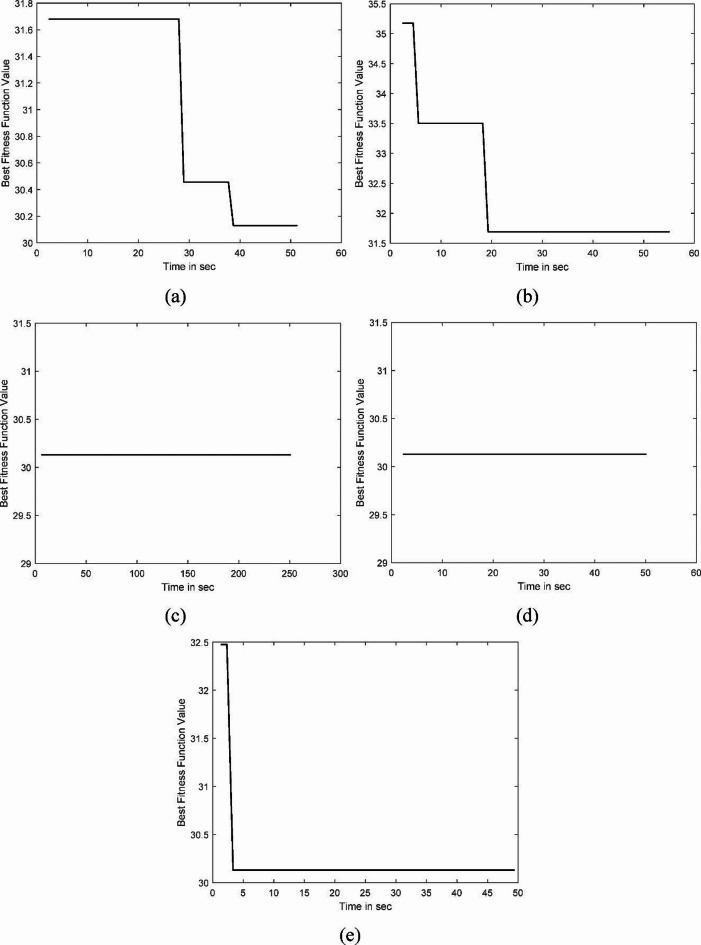
CCs of MOAs on CT slice 1 (**a**) AGDO (**b**) DRO (**c**) IPO (**d**) PWATO (**e**) WOA.

**Fig. 7:**
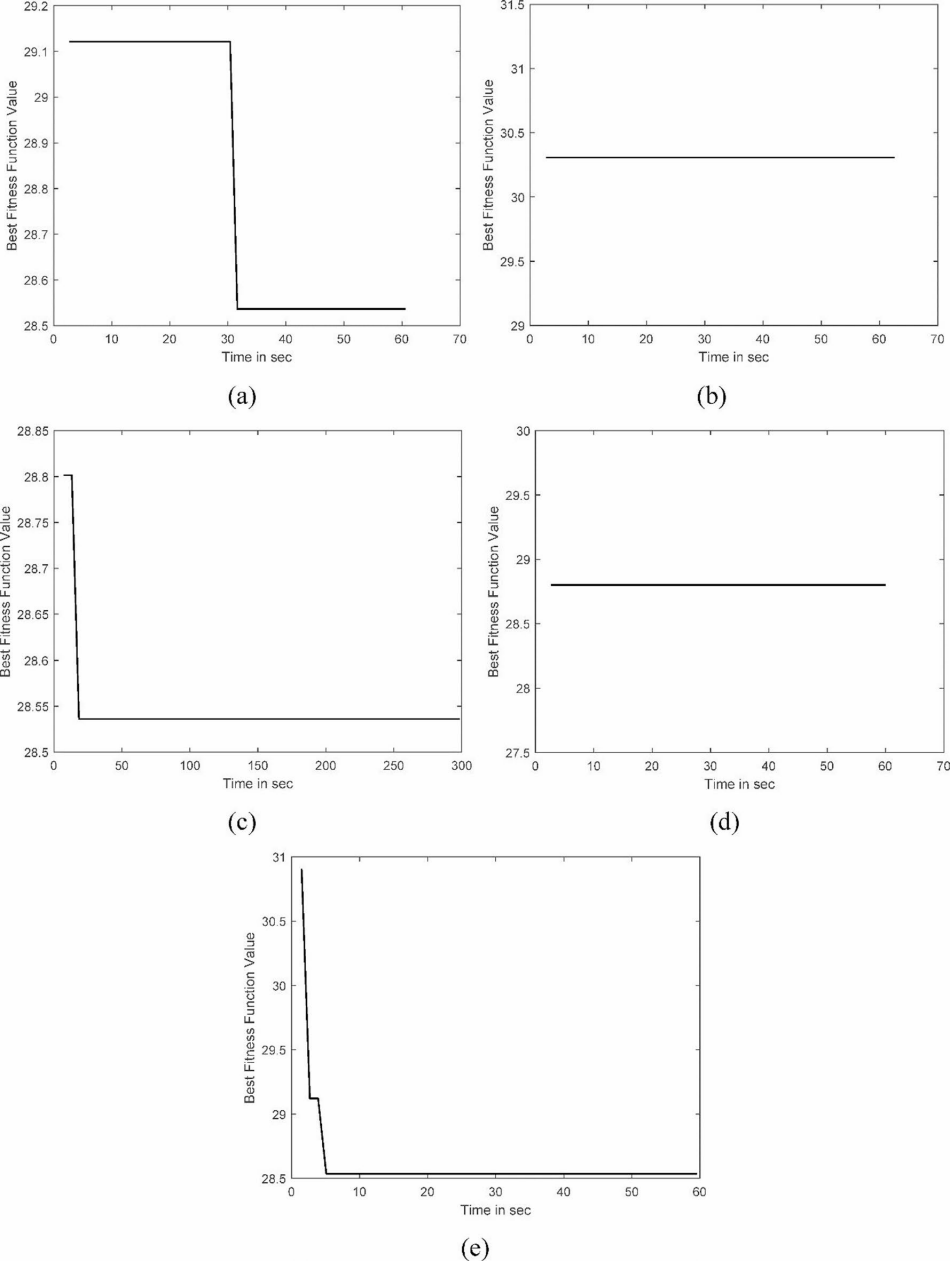
CCs of MOAs on CT slice 2 (**a**) AGDO (**b**) DRO (**c**) IPO (**d**) PWATO (**e**) WOA.

**Fig. 8:**
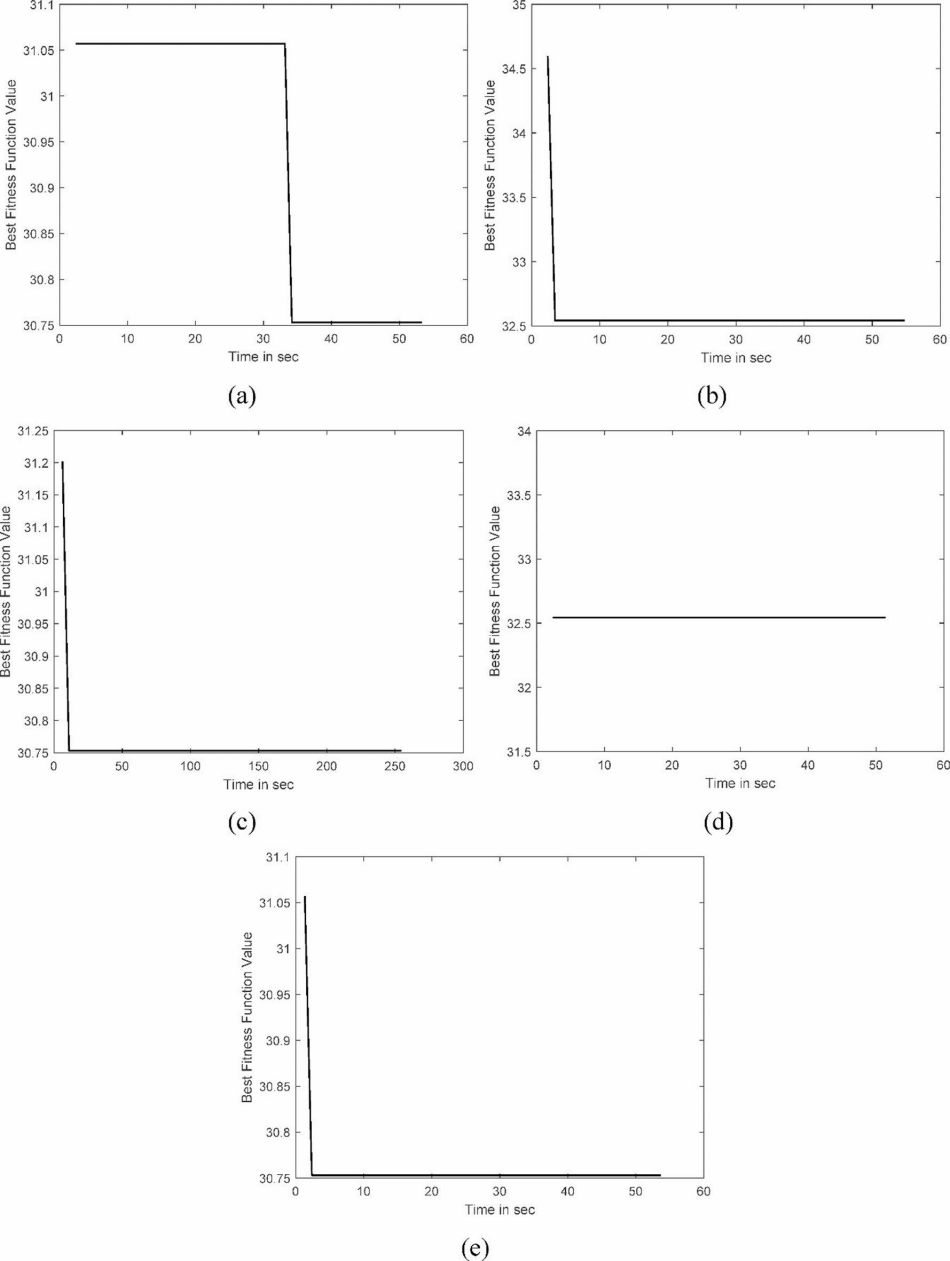
CCs of MOAs on CT slice 3 (**a**) AGDO (**b**) DRO (**c**) IPO (**d**) PWATO (**e**) WO.

We compare the WOA with the latest metaheuristic techniques, namely, Adam gradient descent optimization (AGDO)^21^, divine religions optimizer (DRO)^22^, iterative projection optimization (IPO)^23^, and plant water absorption and transport optimization (PWATO)^24^. Possibility of non-optimal convergence and computational time are two benchmark aspects considered while comparing the WOA with other metaheuristic techniques. Convergence curves (CCs) of these optimizers on three CT slices are shown in Figs. 6, 7 and 8. Minimum PIQE fitness at which these optimizers converge, and corresponding optimum CL values are shown respectively in Tables 1 and 2. Time consumed by the optimizers for convergence is shown in Table 3. Generally, metaheuristic techniques use random initialization of initial position values. Hence, they exhibit inconsistent convergence. Considering this, results reported in Figs. 6, 7 and 8; Tables 1, 2 and 3 are worst-case convergence of the metaheuristic algorithms over 10 repeated trials.

The values in Table 1 that are highlighted in bold are ‘outlier minimum fitness values’ at the convergence of DRO and PWATO. The values in Table 2 that are highlighted in bold are sub-optimal CL supplied by DRO and PWATO. On CT slice 2, DRO gets stuck at the local minimum of the fitness (PIQE) as seen in Fig. 7b. Similarly, on CT slices 2 and 3, PWATO gets stuck at local minima of fitness as seen in Figs. 7d and 8d. AGDO, IPO, and WOA successfully converge to the global minimum of fitness on all test slices. AGDO is computationally slow on all three CT slices, as seen in Table 3. In Table 3, high computational time is highlighted in bold. DRO on slice 1 and IPO on slice 2 are computationally slow. From CCs of the AGDO in Figs. 6a, 7a, and 8a and that of the DRO in Fig. 7b, it can be seen that both AGDO and DRO suffer from the issue of stagnation. Even though they successfully converge to the global minimum of the fitness eventually, they get trapped at a non-minimum fitness for a prolonged time.

**Table 1:** Minimum fitness at which MOAs converge.

Optimization algorithm	CT slice 1	CT slice 2	CT slice 3
AGDO	30.1298	28.5361	30.753
DRO	30.1298	**28.8013**	30.753
IPO	30.1298	28.5361	30.753
PWATO	30.1298	**28.8013**	**32.5422**
WOA	30.1298	28.5361	30.753

**Table 2:** Best CL at which MOAs converge.

Optimization algorithm	CT slice 1	CT slice 2	CT slice 3
AGDO	0.001979	0.002313	0.0023685
DRO	0.0021056	**0.00073725**	0.0022682
IPO	0.0019056	0.002213	0.0023555
PWATO	0.0021568	**0.00053851**	**0.00001**
WOA	0.0019205	0.0023933	0.0024883

**Table 3:** Time consumed by MOAs for convergence in sec.

Optimization algorithm	CT slice 1	CT slice 2	CT slice 3	Observation on 315 CT slices
AGDO	**38.6985**	**31.6414**	**34.1764**	34.8388 ± 3.5749
DRO	**19.3003**	2.7214	3.4321	8.4846 ± 9.3734
IPO	5.9713	**18.3673**	10.9823	11.7736 ± 6.2358
PWATO	2.2645	2.6865	2.3293	2.4268 ± 0.2273
WOA	3.3248	5.0976	2.3633	3.5952 ± 1.3871

### Impact of WOA-PIQE-CLAHE framework on CT quality

**Fig. 9:**
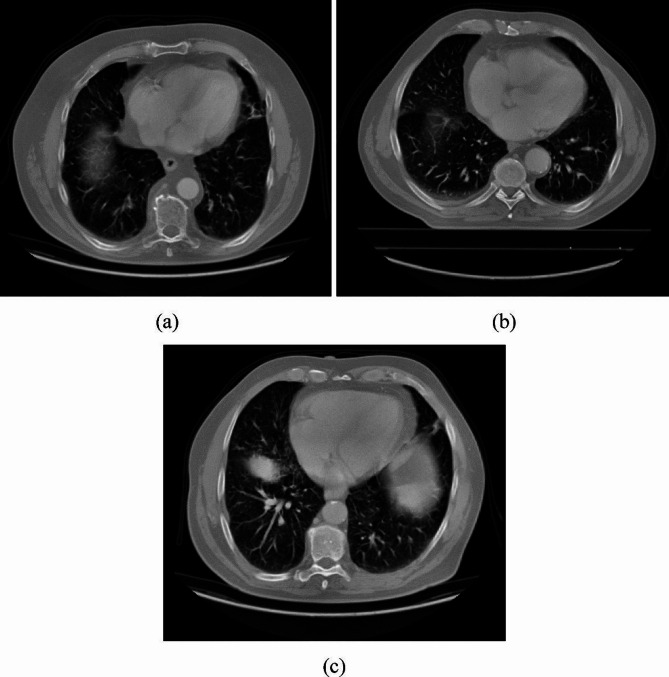
O/Ps of WOA-PIQE-CLAHE framework on representative CT images (**a**) CT slice 1 (**b**) CT slice 2 (**c**) CT slice 3.

If the selection of CL is proper, the CLAHE should improve the tissue contrast without hindering the perceptual quality of the CT slices. Here we manifest that adaptive selection of the CL facilitated by WOA-PIQE framework helps the CLAHE to produce enhanced CT slices high perceptual quality and improved tissue contrast compared to the low-contrast slices via subjective comparisons of perceptual quality of the low-contrast CT and O/Ps of the WOA-PIQE-CLAHE framework and objective analysis of IQAMs like NIQE, PIQE, contrast, and entropy. O/Ps of the WOA-PIQE-CLAHE framework on three representative CT slices (Fig. 3) are shown in Fig. 9. Compared to the original slices in Fig. 3, it is easier for the clinician to discern the structures in the enhanced slices. Tissue contrast is improved without hindering the perceptual quality of the CT slices. From Tables 4 and 5, we can see that O/Ps of the WOA-PIQE-CLAHE framework have lower NIQE and PIQE and higher entropy and contrast compared to the original CT slices. As we try to show improvement of the IQAMs on O/Ps of WOA-PIQE-CLAHE framework with respect to those on low-contrast CT slices, we have not used any IQAs that use both a low-contrast I/P image and an enhanced O/P for computation. Increased contrast and entropy reflect enhanced visibility of structures on the O/Ps of the WOA-PIQE-CLAHE framework. Decreased NIQE and PIQE express improved perceptual quality. These changes in the IQAMs manifest that the WOA-PIQE-CLAHE framework could improve tissue contrast without hindering the perceptual quality of the CT slices.

**Table 4:** No-reference IQA values on low-contrast CT slices.

IQA Metric	CT slice 1	CT slice 2	CT slice 3	Observation on 315 CT slices
NIQE	3.3052	3.0749	3.0302	3.1368 ± 0.1476
PIQE	32.1521	30.9689	32.9341	32.0184 ± 0.9894
Entropy	5.9362	5.7740	6.0527	5.9210 ± 0.1400
Contrast	69.0079	68.4530	70.4782	69.3130 ± 1.0465

**Table 5:** No-reference IQA values of O/Ps of WOA-PIQE-CLAHE framework.

IQA Metric	CT slice 1	CT slice 2	CT slice 3	Observation on 315 CT slices
NIQE	3.0902	2.8133	2.8778	2.9271 ± 0.1449
PIQE	30.1298	28.5361	30.7530	29.8063 ± 1.1433
Entropy	6.2699	6.0536	6.3735	6.2323 ± 0.1632
Contrast	71.1330	70.7614	72.7210	71.5385 ± 1.0408

A metaheuristic framework to facilitate automated selection of the CL in CLAHE for CT contrast enhancement is introduced in the paper. Clinical implications of CLAHE on CT are well established in the literature. Ebenezer et al.^25^ showed that the CLAHE boosts the diagnostic accuracy offered by the EfficientNet in detecting Covid-19 from CT. Similarly, Hrizi et al.^26^ showed that CLAHE increases the segmentation accuracy of lung nodules offered by the swin transformer on CT.

## Conclusion and future scopes

We introduced a metaheuristic framework by combining the whale optimizer and perception-based image quality evaluator (PIQE) fitness to facilitate automated selection of CL of the CLAHE, particularly for contrast enhancement applications in CT. We evaluated the performance of the WOA-PIQE-CLAHE collectively and that of its components individually. We observed that the PIQE possesses the desired attributes of a standard fitness function. The PIQE as a fitness is responsive enough to fine variations of the CL. PIQE has a unique global minimum point for each CT slice. We observed that the WOA is capable of finding out the global minimum of the fitness with minimum computational time and without any issue of stagnation or trapping at the local minima points of the fitness. The framework produces O/Ps that have lower NIQE and PIQE and higher entropy and contrast compared to the original CT slices. WOA-PIQE-CLAHE could improve tissue contrast without hindering the perceptual quality of the CT slices.

The proposed WOA-PIQE-CLAHE framework has certain constraints. PIQE as an image quality metric is particularly sensitive to noise, blur, and blockiness. It is not directly related to the shift in mean brightness caused by the contrast enhancement. The PIQE fitness varies randomly with respect to the variation in CL. Hence, there is an increased probability for the optimizers to get trapped at the local minima points emerging from the random variation. The WOA-PIQE framework facilitates adaptive determination of the CL exclusively for CLAHE used in CT. Further experiments are required to understand the scalability of the framework on other imaging modalities.

## Supplementary Information

Below is the link to the electronic supplementary material.

Supplementary Material 1

## Data Availability

The datasets used and/or analyzed during the current study available from the corresponding author on reasonable request.
